# Comparative analysis between physical activities and circulating lipids from pregnant individuals in Wuhan, from July 2024 to March 2025

**DOI:** 10.3389/fspor.2025.1621665

**Published:** 2025-06-19

**Authors:** Xiaodan He, Yang Liu, Chaoli Chen, Bo Nie

**Affiliations:** ^1^ Department of Laboratory Medicine, Maternal and Child Health Hospital of Hubei Province, Tongji Medical College, Huazhong University of Science and Technology, Wuhan, China; ^2^ Department of Obstetrics, Maternal and Child Health Hospital of Hubei Province, Tongji Medical College, Huazhong University of Science and Technology, Wuhan, China

**Keywords:** physical activity, maternal age, lipid metabolism, pregnancy, maternal health

## Abstract

**Background:**

Maternal circulating lipid concentrations impact the risk of pregnancy complications and infant health outcomes. The associations between physical activity and circulating lipids during pregnancy remain inadequately understood.

**Methods:**

A study was conducted from July 2024 to March 2025, involving the recruitment of 520 pregnant women in Wuhan, China. The Pregnancy Physical Activity Questionnaire (PPAQ) scores were evaluated in trimesters. Circulating lipid profiles, including total triglyceride (TG), total cholesterol (TC), low-density lipoprotein cholesterol (LDL), high-density lipoprotein cholesterol (HDL), apolipoprotein A1 (APOA1) and apolipoprotein B (APOB) concentrations, were assessed at each trimester.

**Results:**

The daily energy expenditure of physical activity (EEPA) during the first, second, and third trimesters was recorded as 11.35, 9.07, and 9.48 metabolic equivalents-hour/day (METs-h/d). The EEPA in the first trimester was significantly greater than that in the second (*p* < 0.001) and third (*p* < 0.01) trimesters. Total daily EEPA levels were significantly elevated in individuals under 35 years old in the first trimester relative to both the second (*p* < 0.01) and third trimesters (*p* < 0.01), whereas no significant difference was found pregnant women over 35 years old. Compared to later trimesters, women in their first trimester exhibited significantly lower circulating levels of TG, TC, LDL, APOA1, and APOB (*p* < 0.0001), but not HDL. Interestingly, while TC, TG, LDL, APOA1, and APOB showed no significant differences across trimesters in older pregnancies, these markers underwent significant changes in younger women. Specifically, TG levels in the younger group increased progressively from the first to the second trimester, whereas no such trend was observed in the older population.

**Conclusion:**

This study suggests that increased physical activity during pregnancy is associated with lower lipid levels. Moreover, maternal age appears to have a significant impact on physical activity and the metabolism of circulating lipids during pregnancy.

## Introduction

Pregnancy is a transformative physiological and psychological period in a woman's life, marked by significant hormonal, metabolic, and cardiovascular adaptations to support fetal development. Pregnant women were often advised to avoid physical exertion due to concerns over potential risks to both mother and baby. However, growing evidence over the past few decades has challenged this notion, demonstrating that regular, moderate physical activity during pregnancy offers substantial benefits with minimal risks when performed appropriately (1). Current guidelines from leading health organizations, including the American College of Obstetricians and Gynecologists (ACOG) and the World Health Organization (WHO), now endorse exercise during pregnancy for women without contraindications, emphasizing its role in enhancing maternal health, improving pregnancy outcomes, and even influencing long-term fetal well-being (2). Engaging in structured physical activity during pregnancy has been linked to a multitude of physiological and psychological advantages. For the mother, exercise can improve cardiovascular fitness, reduce excessive gestational weight gain, and lower the risk of pregnancy-related complications such as gestational diabetes mellitus (GDM), preeclampsia, and venous thromboembolism. Additionally, physical activity has been shown to alleviate common discomforts like lower back pain, pelvic girdle pain, and swelling by enhancing muscular strength, circulation, and posture. Beyond physical benefits, exercise plays a crucial role in mental health, reducing stress, anxiety, and the likelihood of perinatal depression by modulating endorphin and serotonin levels.

Recent studies reveal significant variations in physical activity patterns among pregnant women across different regions. Feng et al. found that only 13.5% of first-time mothers (primiparas) and 15.2% of experienced mothers (multiparas) in Beijing met recommended physical activity guidelines during pregnancy (3). The study demonstrated distinct activity patterns between these groups: while sedentary behavior represented the primary energy expenditure for primiparas when categorized by intensity, household and caregiving activities accounted for most energy expenditure when classified by activity type. Notably, structured exercise contributed minimally to overall energy expenditure. Multiparas generally exhibited higher activity levels across all categories, including total daily activity, light activity, moderate-to-vigorous activity, and household/caregiving tasks (3). Regional comparisons show further disparities. In Guangzhou, pregnant women averaged 17.83 MET-hours of total daily energy expenditure, with a negligible 0.1 MET-hours attributable to exercise. Only 22.6% met international physical activity recommendations (4). A longitudinal study in Hangzhou documented trimester-specific variations, with daily energy expenditure measuring 20.55, 20.76, and 17.19 MET-hours during the first, second, and third trimesters respectively (5). These figures contrast markedly with data from western populations, where pregnant women in Australia and the United States averaged 33.04 and 25.4 MET-hours per day respectively (6, 7). These findings highlight substantial geographical differences in physical activity patterns during pregnancy. However, despite growing regional data, Wuhan remains notably underrepresented in current research. A comprehensive investigation of physical activity behaviors among pregnant women in Wuhan is therefore warranted to complete our understanding of regional variations in maternal activity patterns across China.

In uncomplicated pregnancies, maternal lipid levels demonstrate progressive elevation throughout gestation, with triglyceride (TG) concentrations increasing 2- to 3-fold by the third trimester (8). This physiological hyperlipidemia of pregnancy becomes more pronounced in high-risk pregnancies, where elevated lipid levels have been associated with the pathogenesis of preeclampsia (9, 10). Of particular concern, maternal hypercholesterolemia during pregnancy has been shown to promote fatty streak formation in the fetal aorta, potentially serving as an early marker for future atherosclerotic development and increased cardiovascular disease risk in offspring. Meanwhile, SARS-CoV-2 infection has been reported to disrupt lipid metabolism, particularly affecting HDL and APOA1 (11). Studies have revealed elevated levels of lysophospholipids, triglycerides, sphingomyelins, and oxidized lipids in both maternal and cord plasma following SARS-CoV-2 infection (12). As the initial epicenter of the COVID-19 pandemic, Wuhan experienced widespread SARS-CoV-2 infection among its population during the first outbreak (13). However, there remains a significant research gap regarding how prior SARS-CoV-2 infection affects pregnant women, particularly in terms of its impact on lipid profiles. Current literature lacks comprehensive studies investigating the potential long-term metabolic consequences in this vulnerable demographic.

This study investigates the multifaceted relationship between physical activity, lipids metabolism, and maternal health in pregnant women. Utilizing the PPAQ to assess exercise patterns, we examined comprehensive lipid profiles, including TC, TG, LDL, HDL, APOA1, and APOB. Our analysis synthesizes current evidence to elucidate how optimized physical activity regimens during pregnancy may mitigate potential metabolic disturbances. The findings aim to inform evidence-based prenatal care strategies that support maternal cardiovascular health while promoting long-term wellbeing for both mothers and their children.

## Materials and methods

### Participants recruitment and selection criteria

Pregnant women were conveniently recruited from the Department of Obstetrics at the Maternal and Child Health Hospital of Hubei Province between July 2024 and March 2025. Inclusion criteria participants were eligible if they met the following criteria: gestational age between 12 and 40 weeks (first trimester: 12 weeks to 27 weeks + 6 days; second trimester: 28 weeks to 33 weeks + 6 days; third trimester: ≥34 weeks), singleton pregnancy, and no communication barriers. Participants were excluded if they had physical or mental conditions restricting normal activity, incomplete clinical data, and cardiac, hepatic, or hematologic disorders complicating pregnancy.

### Pregnant physical activity questionnaire details

The Pregnant Physical Activity Questionnaire (PPAQ) was used to assess physical activity (PA) during pregnancy. Originally developed by Chasan-Taber (14) in 2004, it was later translated into Chinese by Zhang et al. in 2012 (15). The questionnaire primarily measures the duration, frequency, and intensity of activities performed during pregnancy. The PPAQ consists of 31 items, categorized into four domains based on activity type: Household/caregiving activities (14 activities), Outdoor activities (4 activities), Sports/exercise (8 activities), Occupational activities (5 activities). Activity intensity was classified according to metabolic equivalents (METs) as follows: sedentary (<1.5 METs), light intensity (1.5–2.9 METs), moderate intensity (3.0–6.0 METs), Vigorous intensity (>6.0 METs). Additionally, activities were divided into six different levels based on duration and frequency, each with a corresponding time-weight coefficient. To calculate each participant's energy expenditure, the frequency of different physical activities was multiplied by their respective time-weight coefficients and energy consumption values. The sum of these values represented the participant's total energy expenditure, with higher values indicating greater physical activity during pregnancy. The questionnaire demonstrated strong psychometric properties, with a content validity index of 0.940 and a test-retest reliability of 0.940. In this study, adherence to exercise guidelines was determined based on the American Congress of Obstetricians and Gynecologists (ACOG) recommendations for physical activity during pregnancy (16). Participants who engaged in ≥7.5 MET-hours per week of moderate or higher-intensity exercise (at least 5 days per week, 30 min per day) were considered to have met the exercise standard.

### Study population and data collection

Data of PPAQ details, including age, pregnant duration, residence, occupation, and degree of education were obtained from each enrolled patient. Clinical signs, laboratory test results of circulating lipids (AU5800, Beckman) were retrieved from electronic medical records of the Hospital Authority. This study was approved by the Medical Ethics Committee of the Maternal and Child Health Hospital of Hubei Province (2025-092-01).

### Statistical analysis

Data were collected and managed in Microsoft Excel by two independent investigators. Statistical analyses were performed using SPSS 27.0 (IBM Corp.). Normally distributed continuous variables were reported as mean ± standard deviation (SD). Non-normally distributed data were expressed as median and interquartile range (IQR). Categorical variables were summarized as frequencies and percentages. Differences in physical activity levels across time points were assessed using either analysis of variance (ANOVA) or the Friedman rank-sum test, as appropriate. A two-sided *P*-value < 0.05 was considered statistically significant.

## Results

### General information of the participants

This study included over 520 pregnant women across all three trimesters. Participants independently completed the Pregnancy Physical Activity Questionnaire (PPAQ). However, some of them provided incomplete PPAQ data. Additionally, due to pregnancy complications limiting physical activity or lipid testing being conducted at other hospitals, a subset of participants did not complete follow-up lipid examinations. Ultimately, 520 participants met the inclusion criteria and were included in the final statistical analysis. This study administered women in the first trimester (29%), second trimester (32%), and third trimester (39%) of pregnancy. Table 1 presents the demographic characteristics of the study participants.

**Table 1:** Demographic characteristics of the participants.

Category	Number	Percentage (%)
Age (years)		
First trimester	151	29
<35	123	81
≥35	28	19
Second trimester	164	32
<35	128	78
≥35	36	22
Third trimester	205	39
<35	159	76
≥35	46	24
Residence		
Town	442	85
Country	78	15
Occupation		
Employed	371	71
Full-time mom	149	29
Degree of education		
Junior and below	37	7
High school	51	9
Junior college	176	34
Undergraduate	199	38
Master's degree of above	57	12

### Energy expenditure of physical activity (EEPA) across pregnancy trimesters

**Figure 1:**
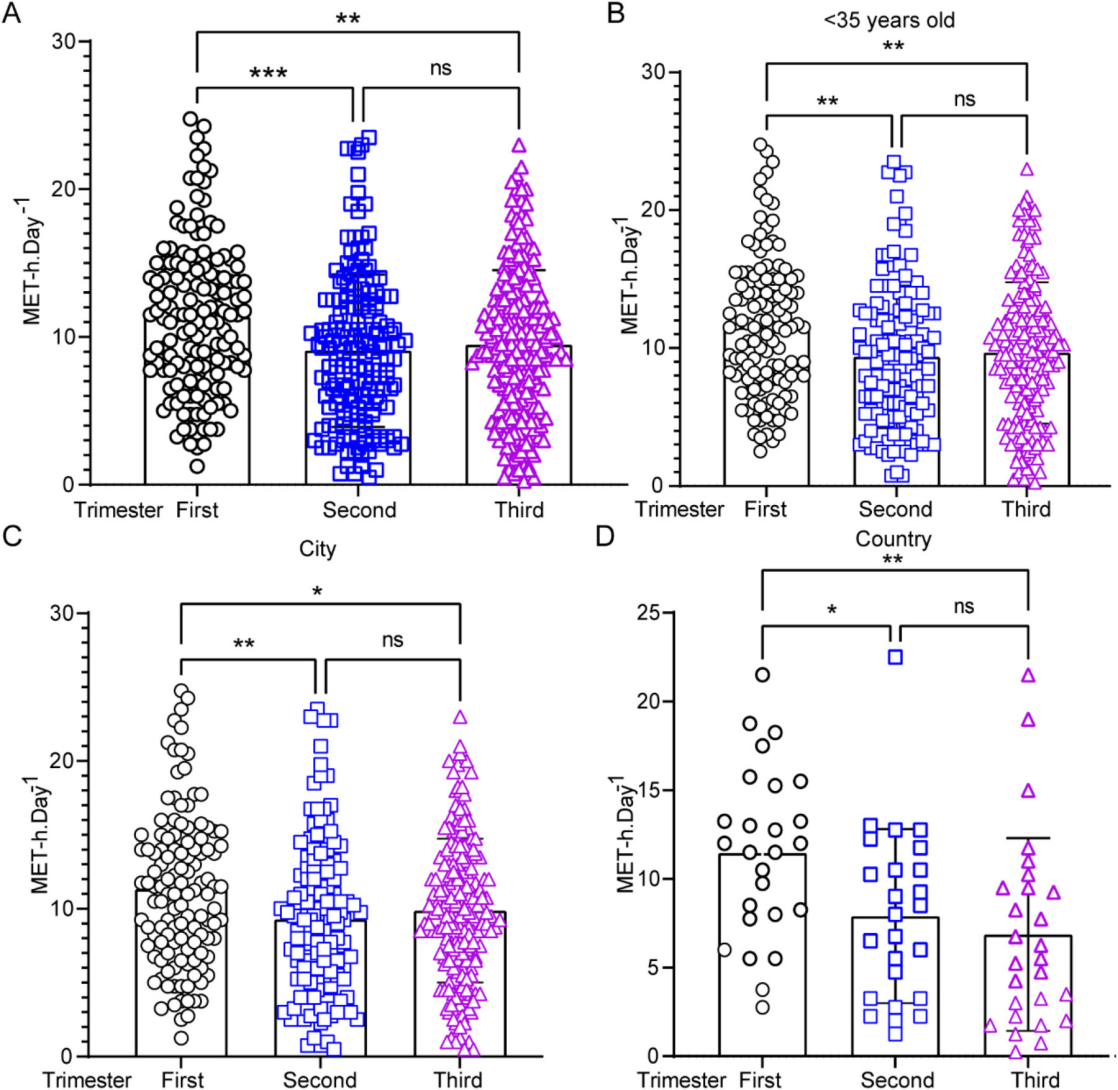
Energy expenditure of physical activity (EEPA) during pregnancy: variations by trimester, age, and region. **(A)** EEPA analysis in the first (*n* = 151), second (*n* = 164), and third (*n* = 205) trimesters. Data were analyzed by one-way ANOVA (***, *p* < 0.001; **, *p* < 0.01). **(B–D)** Comparison of EEPA of pregnant women under 35 years old in the first, second, and third trimesters. **(C,D)** Statistical EEPA analysis of pregnant women from city **(C)** or country **(D)** through trimesters. Statistical significance was determined by Student's t-test (**, *p* < 0.01; *, *p* < 0.05; ns = not significant, *p* > 0.05).

The total EEPA during the first, second, and third trimesters of pregnancy was measured as follows: first trimester (*n* = 151, 11.35 ± 5.05 METs-h/d), second trimester (*n* = 164, 9.07 ± 5.15 METs-h/d), and third trimester (*n* = 205, 9.48 ± 5.04 METs-h/d) (Figure 1A). Repeated-measures ANOVA revealed a statistically significant difference in total daily EEPA across trimesters (*F* = 9.074, *p* < 0.001). *post-hoc* comparisons showed that total daily EEPA was significantly higher in the first trimester than in both the second (*t* = 2.281, *p* < 0.001) and third trimesters (*t* = 1.874, *p* < 0.01). However, no significant difference was observed between the second and third trimesters (*t* = −0.407, *p* > 0.05). To evaluate whether maternal age influences EEPA, we compared total EEPA between participants under 35 years (<35 years) and those 35 years or older (≥35 years) across pregnancy trimesters. The analysis revealed a more pronounced decline in EEPA throughout pregnancy among participants under 35 years old (Figure 1B). In this group, EEPA was significantly higher in the first trimester compared to both the second (*t* = 2.137, *p* < 0.01) and third trimesters (*t* = 1.843, *p* < 0.01). In contrast, this declining trend was not observed in participants aged 35 or older (Supplementary Figure S1A). A similar decline in physical activity was observed in both urban (Figure 1C) and rural (Figure 1D) participants. Further analysis indicated that urban pregnant women in the third trimester exhibited higher EEPA levels compared to their rural counterparts (Supplementary Figure S1B–D). These findings suggest that total EEPA decreases as pregnancy progresses, particularly in individuals under 35 years of age. Surprisingly, maternal age over 35 years old was associated with lower EEPA during all the pregnancy trimesters.

### Longitudinal changes in lipid profiles across pregnancy trimesters

**Figure 2:**
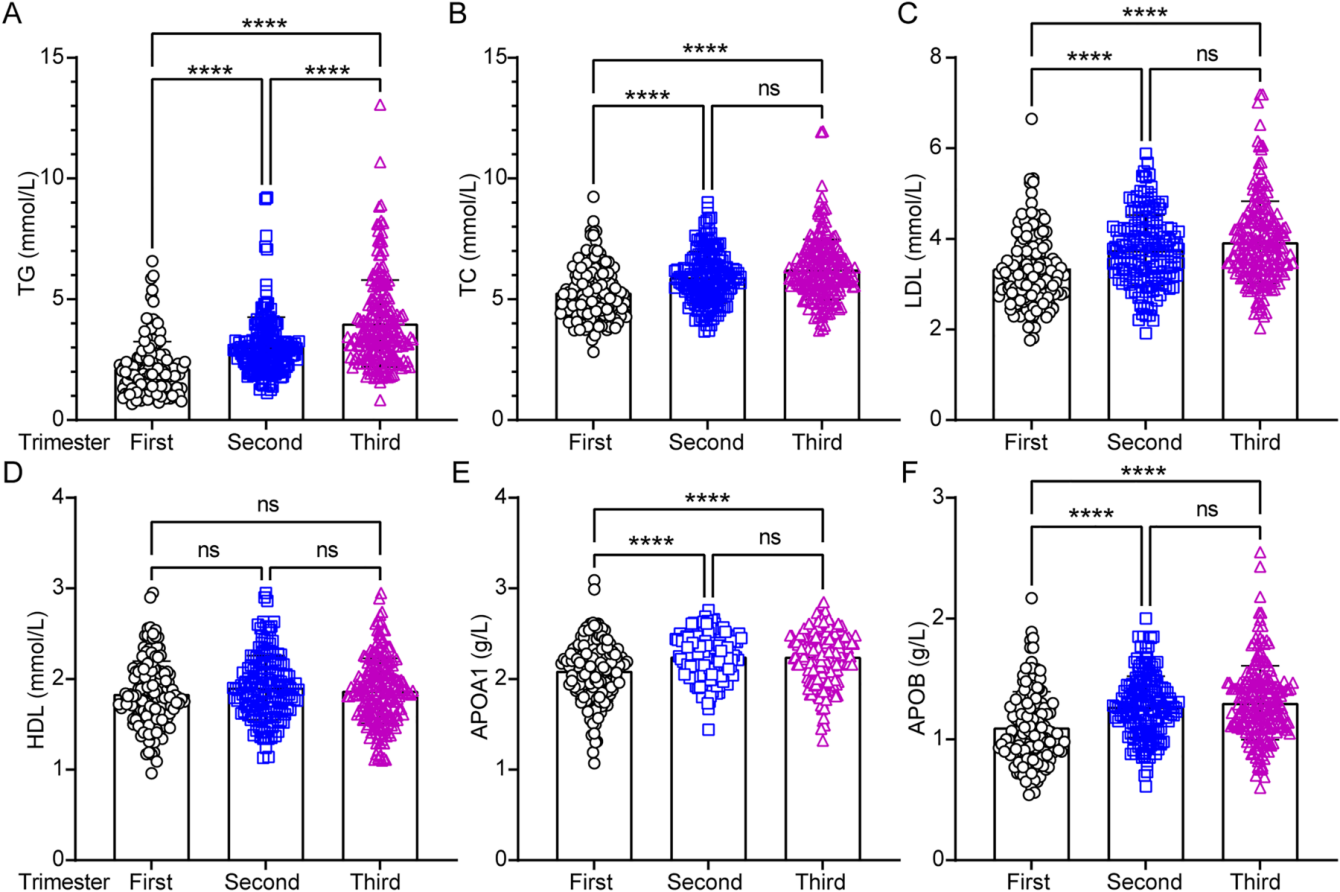
Analysis of circulating lipids profiles across the pregnancy trimesters. Serum lipid parameters including total triglyceride (TG), total cholesterol (TC), low-density lipoprotein cholesterol (LDL), high-density lipoprotein cholesterol (HDL), apolipoprotein A1 (APOA1) and apolipoprotein B (APOB) were measured and statistically analyzed across trimesters. Data were analyzed by one-way ANOVA (****, *p* < 0.0001; ns = not significant, *p* > 0.05).

Circulating lipid levels were analyzed during the first, second, and third trimesters of pregnancy. Women with high EEPA exhibited significantly lower first-trimester levels of TG (Figure 2A; *p* < 0.0001), TC (Figure 2B; *p* < 0.0001), and LDL (Figure 2C; *p* < 0.0001) compared to those with low EEPA. Additionally, lipoprotein levels specifically APOA1 (Figure 2E; *p* < 0.0001) and APOB (Figure 2F; *p* < 0.0001) were lower in the first trimester than in the second and third trimesters. While TG levels continued to rise from the second to the third trimester, no significant changes were observed in TC, LDL, HDL, APOA1, or APOB during this period (Figure 2A). Notably, circulating HDL levels remained stable across all three trimesters, with no significant variations (Figure 2D).

**Figure 3:**
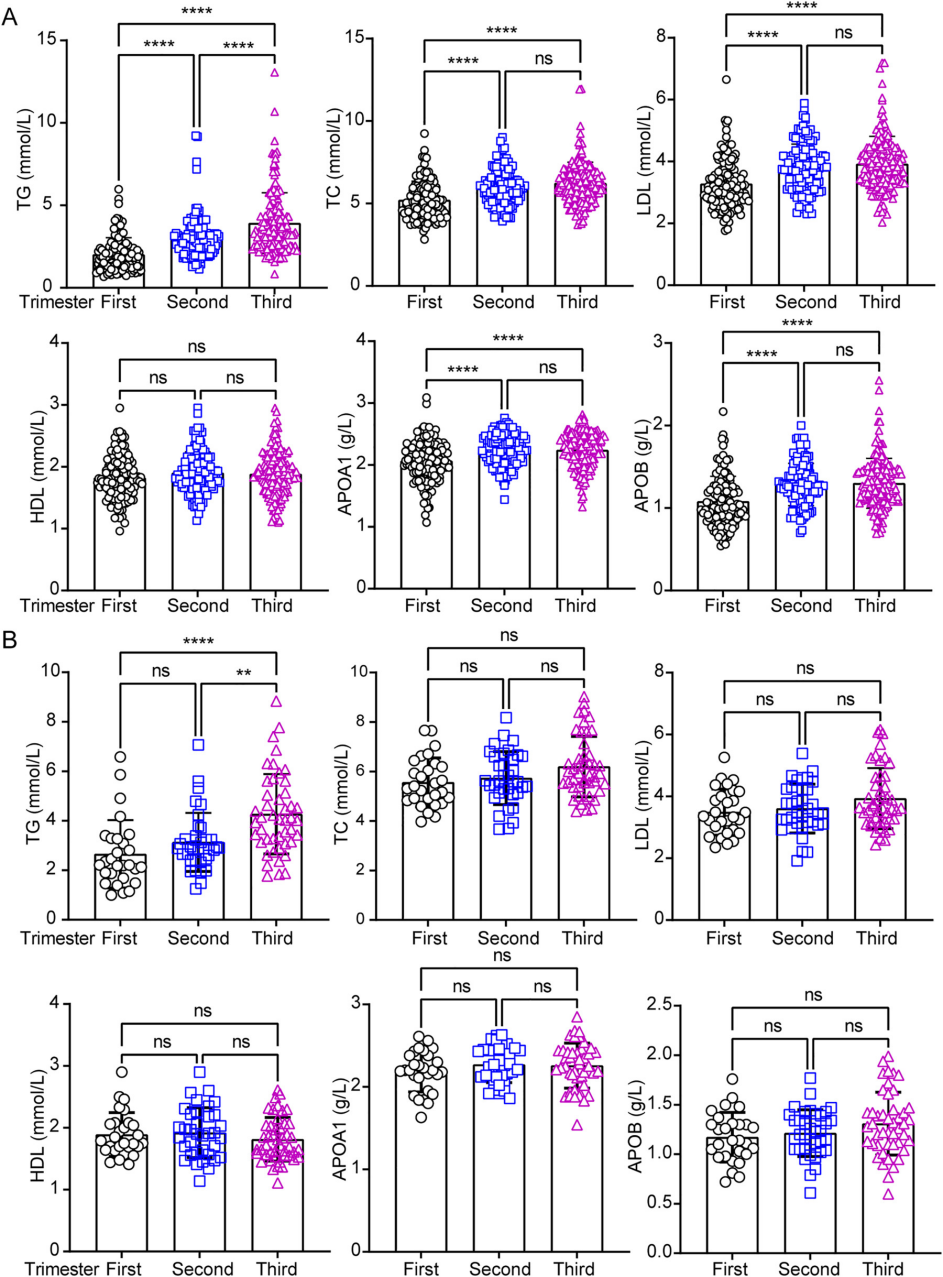
Analysis of circulating lipids profiles across the pregnancy trimesters in under and over 35-years old pregnant women. The circulating lipids including total triglyceride (TG), total cholesterol (TC), low-density lipoprotein cholesterol (LDL), high-density lipoprotein cholesterol (HDL), apolipoprotein A1 (APOA1) and apolipoprotein B (APOB) were performed statistical analysis separately in under **(A)** and over 35-years **(B)** pregnant women in three trimesters. Data were analyzed by one-way ANOVA with the following significance levels: ****, *p* < 0.0001; **, *p* < 0.01; ns = not significant (*p* > 0.05).

To investigate whether maternal age alters circulating lipid profiles during pregnancy, this study revealed only similar trends in circulating HDL level between the two groups throughout pregnancy (Figures 3A,B). However, while circulating TC, LDL, APOA1, and APOB showed no significant differences in pregnancies aged 35 or older (Figure 3B), it exhibited significant changes in participants under 35 years old (Figure 3A). In addition, TG levels continued to rise from the first to the second trimester in pregnancies under 35 years old (Figure 3A), whereas no significant changes were observed in their counterparts during the same period (Figure 3B). To assess whether circulating lipid profiles differ between age-dependent pregnancies across trimesters, we compared lipid levels in each period. The results revealed no significant differences in any trimesters or between groups, except TG in the first trimester (Supplementary Figure S2). To further analyze the correlation between PA and lipid profiles, scatter plots were used to assess the relationship between PA and circulating lipid parameters, including TG, TC, LDL, HDL, APOA1, and APOB. A significant negative correlation was observed between PA and APOB levels (Table 2; *p* < 0.05), indicating that higher physical activity was associated with lower APOB concentrations. In contrast, no significant association was detected between PA and TC, TG, LDL, HDL, APOA1 (Table 2; *p* > 0.05). These results highlight the potential benefits of physical activity in modulating lipid metabolism, particularly in reducing atherogenic lipid APOB.

**Table 2:** Analysis of influencing factors of PA during pregnancy.

Category	TG	TC	LDL	HDL	APOA1	APOB
Pearson correlation	−0.084	−0.047	−0.064	0.042	0.001	−0.090^a^
Sig. (2-tailed)	0.055	0.281	0.142	0.334	0.977	0.040
Sum of Squares and Cross-products	−369.88	−154.79	−150.03	40.666	0.980	−72.393
Covariance	−0.713	−0.298	−0.289	0.078	0.002	−0.139
95% CI (Upper limit)	−0.003	0.030	0.009	0.135	0.082	−0.018
95% CI (Lower limit)	−0.163	−0.128	−0.147	−0.040	−0.085	−0.170

^a^ Correlation is significant at the 0.05 level (2-tailed).

These findings suggest that maternal age is associated with distinct lipid profile patterns in pregnancy compared to counterpart pregnancies. Specifically, TG levels further increased in the second trimester among pregnancies under 35 years old, and TC, LDL, APOA1, and APOB displayed divergent profiles between the two groups.

## Discussion

This study evaluated changes in physical activity, circulating lipid profiles among pregnant women in Wuhan. We found that the total daily physical activity energy expenditure during the first, second, and third trimesters was approximately 11.35, 9.07, and 9.48 METs-h/d, respectively, slightly lower than values reported in surveys from Beijing (3), Guangzhou (4) and Hangzhou (5). Furthermore, compared to pregnant women in Australia (30.0 METs-h/d) (6) and the United States (25.4 METs-h/d) (7), physical activity levels among Chinese pregnant women were substantially lower throughout pregnancy. Regarding trends in physical activity changes, the second and third trimesters exhibited similar levels, while a significant increase was observed in the first trimester. This finding aligns with results from Zhang et al. in their study of pregnant women in Hangzhou (5). The higher activity in the first trimester may reflect traditional health behaviors, as this period is critical for fetal growth and development, prompting more active lifestyles. By the second trimester, as fetal growth stabilizes, physical activity tends to decline. Interestingly, despite increased fatigue and discomfort in the third trimester due to greater bodily weight, physical activity showed a slight rebound compared to the second trimester. Multiple studies (17) suggest that moderate exercise during late pregnancy can alleviate discomfort, strengthen pelvic muscles and ligaments, and facilitate natural delivery.

Interestingly, we observed an age-related difference. Women under 35 years old demonstrated greater physical activity during the first trimester. No significant trimester-to-trimester variations in activity levels were found among women over 35 years old. These findings may be attributable to traditional health behaviors that vary by age group. Variations in occupational status and educational attainment may also influence physical activity patterns (18, 19). Notably, this study revealed that pregnant women in the city exhibited higher physical activity levels during the third trimester compared to their counterparts, though no such difference was observed in the first and second trimester. This pattern may reflect heightened health awareness among urban individuals. These findings highlight the need to promote awareness of proper and regular physical activity during pregnancy in China. Public health efforts in Wuhan should emphasize the benefits of prenatal exercise, encouraging and guiding pregnant women, especially over 35 years old population, to engage in safe, appropriate physical activity.

During pregnancy, there is a progressive increase in plasma TG, TC, and lipoprotein levels including HDL and LDL (20, 21). These physiological adaptations in lipid metabolism ensure a continuous nutrient supply to support fetal growth (20). However, SARS-CoV-2 infection has been reported to disrupt lipid metabolism, particularly affecting HDL and APOA1 (11). Studies have revealed elevated levels of lysophospholipids, triglycerides, sphingomyelins, and oxidized lipids in both maternal and cord plasma following SARS-CoV-2 infection (12). Consistent with prior findings, this study observed similar trends in TC, TG, and LDL levels. Notably, HDL concentrations remained stable throughout all pregnancy, whereas in women under 35 years old, TC, TG, LDL, APOA1 and APOB levels increased significantly in the second and third trimesters. This pattern couldn't be observed in pregnant populations over 35 years old (11). Meanwhile, HDL levels showed no significant variations, regardless of age or trimester. An important finding was the sustained rise in TG levels across all trimesters in young pregnant women, whereas elder cohorts exhibited no significant difference between the first and second trimesters. Excessive TG accumulation may heighten maternal health risks and adversely affect newborns. Interestingly, although absolute lipid concentrations did not differ significantly between young and elder groups within the same trimester, a higher proportion of women over 35 years old had displayed abnormal lipid levels in the first trimester. Even slightly elevated results may pose disproportionate risks to both mothers and infants. Whether and how SARS-CoV-2 infection affect maternal PA and lipid metabolism need further investigation. In conclusion, this study underscores that maternal age is a critical factor influencing physical activity and lipid metabolism, thereby potentially affecting maternal and fetal safety.

## Data Availability

The original contributions presented in the study are included in the article/Supplementary Material, further inquiries can be directed to the corresponding author.

## References

[1] Ribeiro MM Andrade A Nunes I. Physical exercise in pregnancy: benefits, risks and prescription. J Perinat Med. (2022) 50:4–17. 10.1515/jpm-2021-0315 34478617

[2] Practice, A.C.O. ACOG Committee opinion. Number 267, January 2002: exercise during pregnancy and the postpartum period. Obstet Gynecol. (2002) 99:171–3. 10.1097/00006250-200201000-00030 11777528

[3] Li J Zou F Huang S Zhai J Cai W. Cognition on exercise of pregnant women between primiparas and multiparas during pregnancy and their prenatal physical activities. Chin Gen Pract. (2016) 19:3983–6. 10.3969/j.issn.1007-9572.2016.32.017

[4] Yang H Deng Y Gao L. Current status and influencing factors of physical activity in pregnant women during pregnancy in Guangzhou. Chin Evid Based Nurs. (2017) 3:238–43. 10.3969/j.issn.2095-8668.2017.03.012

[5] Zhang L Piao J Zhang W Liu N Zhang X Shen Y Physical activity changes and influencing factors among Chinese pregnant women: a longitudinal study. J Matern Fetal Neonatal Med. (2024) 37:2306190. 10.1080/14767058.2024.2306190 38262926

[6] Schmidt MD Pekow P Freedson PS Markenson G Chasan-Taber L. Physical activity patterns during pregnancy in a diverse population of women. J Womens Health (Larchmt). (2006) 15(8):909–18. 10.1089/jwh.2006.15.909 17087614

[7] Evenson KR Wen F. Prevalence and correlates of objectively measured physical activity and sedentary behavior among US pregnant women. Prev Med. (2011) 53:39–43. 10.1016/j.ypmed.2011.04.014 21575654

[8] Dempsey JC Williams MA Leisenring WM Shy K Luthy DA. Maternal birth weight in relation to plasma lipid concentrations in early pregnancy. Am J Obstet Gynecol. (2004) 190:1359–68. 10.1016/j.ajog.2003.10.710 15167842

[9] Clausen T Djurovic S Henriksen T. Dyslipidemia in early second trimester is mainly a feature of women with early onset pre-eclampsia. BJOG. (2001) 108:1081–7. 10.1111/j.1471-0528.2001.00247.x 11702841

[10] Samsuddin S Arumugam PA Md Amin MS Yahya A Musa N Lim LL Maternal lipids are associated with newborn adiposity, independent of GDM status, obesity and insulin resistance: a prospective observational cohort study. BJOG. (2020) 127:490–9. 10.1111/1471-0528.16031 31778255

[11] Hilser JR Han Y Biswas S Gukasyan J Cai Z Zhu R Association of serum HDL-cholesterol and apolipoprotein A1 levels with risk of severe SARS-CoV-2 infection. J Lipid Res. (2021) 62:100061. 10.1016/j.jlr.2021.100061 33667465 PMC7923911

[12] Frankevich N Tokareva A Chagovets V Starodubtseva N Dolgushina N Shmakov R COVID-19 infection during pregnancy: disruptions in lipid metabolism and implications for newborn health. Int J Mol Sci. (2023) 24:13787. 10.3390/ijms241813787 37762087 PMC10531385

[13] Huang C Wang Y Li X Ren L Zhao J Hu Y Clinical features of patients infected with 2019 novel coronavirus in Wuhan, China. Lancet. (2020) 395:497–506. 10.1016/S0140-6736(20)30183-5 31986264 PMC7159299

[14] Chasan-Taber L Schmidt MD Roberts DE Hosmer D Markenson G Freedson PS. Development and validation of a pregnancy physical activity questionnaire. Med Sci Sports Exerc. (2004) 36:1750–60. 10.1249/01.MSS.0000142303.49306.0D 15595297

[15] Xiang M Konishi M Hu H Takahashi M Fan W Nishimaki M Reliability and validity of a Chinese-translated version of a pregnancy physical activity questionnaire. Matern Child Health J. (2016) 20:1940–7. 10.1007/s10995-016-2008-y 27112554

[16] Committee on Obstetric Practice. Physical activity and exercise during pregnancy and the postpartum period: ACOG committee opinion, number 804. Obstet Gynecol. (2020) 135:e178–88. 10.1097/AOG.0000000000003772 32217980

[17] Sanabria-Martinez G Poyatos-Leon R Notario-Pacheco B Alvarez-Bueno C Cavero-Redondo I Martinez-Vizcaino V. Effects of physical exercise during pregnancy on mothers’ and neonates’ health: a protocol for an umbrella review of systematic reviews and meta-analysis of randomised controlled trials. BMJ Open. (2019) 9:e030162. 10.1136/bmjopen-2019-030162 31519677 PMC6747876

[18] Rodrigues-Denize N Zolnikov BTR Furio F. A systematic review on the physical, mental, and occupational effects of exercise on pregnant women. Dialogues Health. (2024) 4:100181. 10.1016/j.dialog.2024.100181 38813580 PMC11133494

[19] Magro Malosso ER Saccone G Di Mascio D Berghella V. Maternal education predicts compliance to exercise during pregnancy. Acta Obstet Gynecol Scand. (2019) 98:809. 10.1111/aogs.13550 30698274

[20] Butte NF. Carbohydrate and lipid metabolism in pregnancy: normal compared with gestational diabetes mellitus. Am J Clin Nutr. (2000) 71(5 Suppl):1256S–61. 10.1093/ajcn/71.5.1256s 10799399

[21] Sattar N Greer IA Louden J Lindsay G McConnell M Shepherd J Lipoprotein subfraction changes in normal pregnancy: threshold effect of plasma triglyceride on appearance of small, dense low density lipoprotein. J Clin Endocrinol Metab. (1997) 82:2483–91. 10.1210/jcem.82.8.4126 9253322

