# Supplementary material for: Comparative analysis between physical activities and circulating lipids from pregnant individuals in Wuhan, from July 2024 to March 2025
Source: Front Sports Act Living. 2025 Jun 19;7:1621665. doi: 10.3389/fspor.2025.1621665 (PMC12224188; doi:10.3389/fspor.2025.1621665)
Supplement: Supplementary file 1 [file Datasheet1.docx]

Supplementary Figures


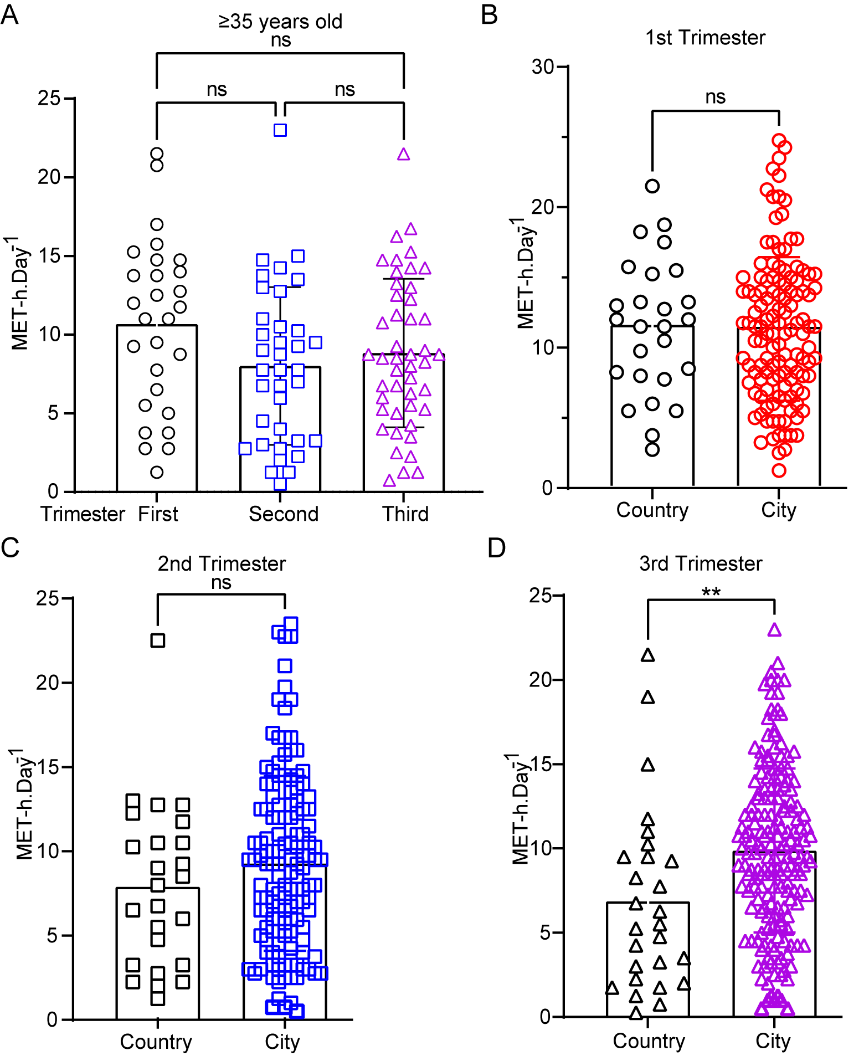


Supplementary Figure 1. Age and region variations in physical activity patterns

The analysis of energy expenditure of physical activity (EEPA) in participants over 35 years old pregnant women (A). The difference of EEPA between city and country populations exposed in the first (B), second (C), and third (D) trimesters. Data were analyzed by one-way ANOVA (**, p<0.01; ns=not significant, p>0.05).


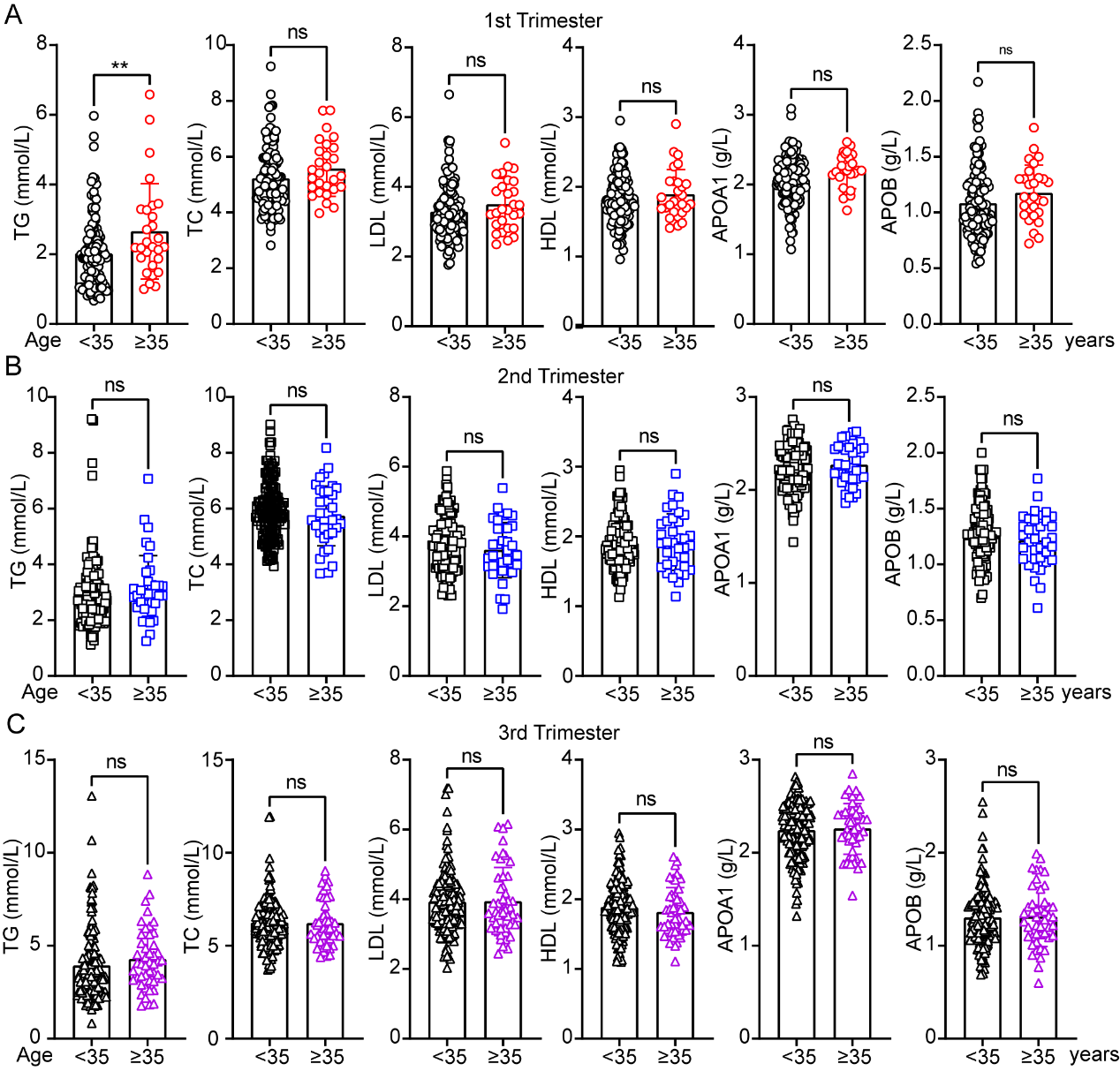


Supplementary Figure 2. Analysis of lipid profiles across pregnancy trimesters by age.

The analysis of the circulating lipids including total triglyceride (TG), total cholesterol (TC), low-density lipoprotein cholesterol (LDL), high-density lipoprotein cholesterol (HDL), apolipoprotein A1 (APOA1) and apolipoprotein B (APOB) in the first (A), second (B), third (C) trimesters between under- or over- 35 years old pregnant women. Data were analyzed by one-way ANOVA (ns=not significant, p>0.05).


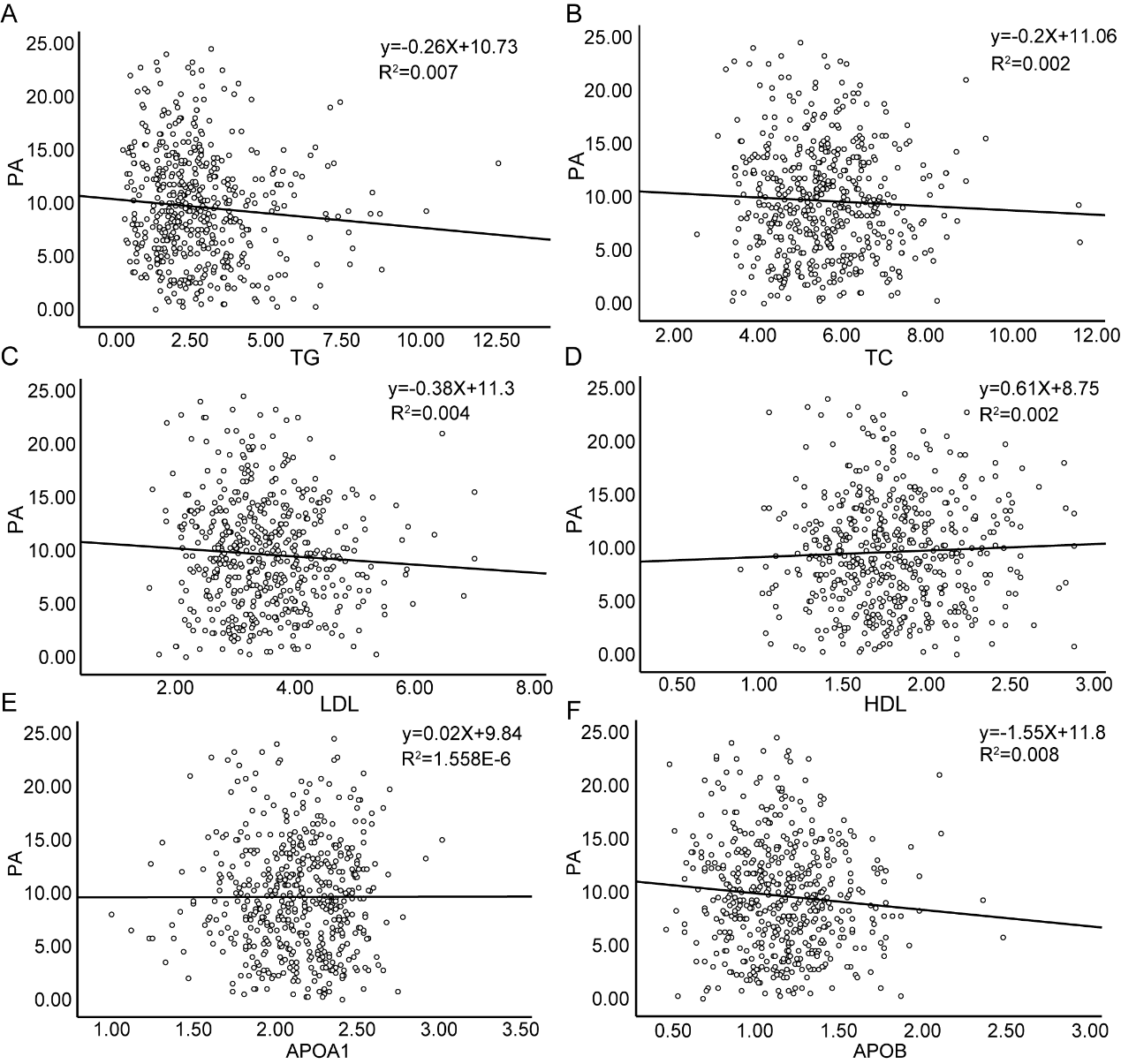


Supplementary Figure 3. The correlation analysis between PA and lipid profiles in pregnant women.

A-F. Scatter plots depict the relationship between physical activity (PA) and lipid parameters including total triglyceride (A), total cholesterol (B), low-density lipoprotein cholesterol (C), high-density lipoprotein cholesterol (D), apolipoprotein A1 (E) and apolipoprotein B (F). Correlation coefficients (R^2^) and p-values are indicated where significant.
